# Supplementary material for: Drugs That Induce Gingival Overgrowth Drive the Pro-Inflammatory Polarization of Macrophages In Vitro
Source: Int J Mol Sci. 2024 Oct 24;25(21):11441. doi: 10.3390/ijms252111441 (PMC11546752; doi:10.3390/ijms252111441)
Supplement: Supplementary file 1 [file ijms-25-11441-s001.zip › ijms-3254639-supplementary/Table S1.pdf]

Table S1. Gene expression profile in differentiated macrophage subtypes. M0 macrophages treated with LPS and IFNγ showed the overexpression of the M1 markers; M0 macrophages treated with IL-4 showed the overexpression of the M2 markers.

Gene	LPS + IFNγ		IL4	
	**Fold change**	***p* value**	**Fold change**	***p* value**
CCL5	**53.28**	**0.010**	1.14	0.226
CXCL10	**18.03**	**0.017**	0.27	0.028
IDO1	**14.54**	**0.020**	0.31	0.008
CD23	1.45	0.246	**40.66**	**0.006**
MRC1	0.29	0.020	**25.50**	**0.002**
CCL22	1.48	0.078	**73.71**	**0.008**

In bold significant change in gene expression level

Significantly up-regulated gene: fold change ≥ 2 (p value ≤ 0.05)

Significantly down-regulated gene: fold change ≤ 0.5 (p value ≤ 0.05)
