# Supplementary material for: Drugs That Induce Gingival Overgrowth Drive the Pro-Inflammatory Polarization of Macrophages In Vitro
Source: Int J Mol Sci. 2024 Oct 24;25(21):11441. doi: 10.3390/ijms252111441 (PMC11546752; doi:10.3390/ijms252111441)
Supplement: Supplementary file 1 [file ijms-25-11441-s001.zip › ijms-3254639-supplementary/Table S2.pdf]

Table S2. Gene expression profile of polarization markers of macrophages after 24h drug exposure.

Gene	Mycophenolate 10 µM		Gabapentin 100 µM		Amlodipine 1 µM		Diphenylhydantoin 10 µM	
	**Fold change**	***p* value**	**Fold change**	***p* value**	**Fold change**	***p* value**	**Fold change**	***p* value**
CCL5	**2.47**	**0.036**	**4.13**	**0.033**	**2.85**	**0.021**	**3.37**	**0.036**
CXCL10	**5.70**	**0.005**	**7.00**	**0.002**	**4.16**	**0.032**	**4.96**	**0.017**
IDO1	**4.98**	**0.018**	**7.72**	**0.033**	**7.37**	**0.013**	**9.60**	**0.026**
CD23	1.16	0.257	1.91	0.188	1.11	0.638	1.49	0.121
MRC1	1.07	0.119	1.72	0.047	1.84	0.272	1.43	0.121
CCL22	0.80	0.563	1.50	0.391	1.36	0.208	1.36	0.238

In bold significant change in gene expression level

Significantly up-regulated gene: fold change ≥ 2 (*p* value ≤ 0.05)

Significantly down-regulated gene: fold change ≤ 0.5 (*p* value ≤ 0.05)
